# Digital pathology-based artificial intelligence model to predict microsatellite instability in gastroesophageal junction adenocarcinomas

**DOI:** 10.3389/fonc.2025.1486140

**Published:** 2025-08-07

**Authors:** Zhenqian Li, JingQi Chen, Miaomiao Sun, Daoming Li, Kuisheng Chen

**Affiliations:** ^1^ Department of Pathology, the First Affiliated Hospital of Zhengzhou University, Zhengzhou, China; ^2^ Department of Clinical Medicine, Mudanjiang Medical University, Mudanjiang, China

**Keywords:** artificial intelligence, deep machine learning, gastroesophageal junction adenocarcinomas, digital pathology, microsatellite instability, microsatellite instability high

## Abstract

**Purpose:**

Microsatellite instability (MSI) plays a crucial role in determining the therapeutic outcomes of gastroesophageal junction (GEJ) adenocarcinoma. This study aimed to develop a deep learning model based on H&E-stained pathological specimens to accurately identify MSI-H in GEJ adenocarcinomas patients.

**Methods:**

A total of 416 H&E-stained slides of 212 GEJ adenocarcinoma patients were collected to establish an artificial intelligence (AI) model using digital pathology (DP) for of MSI-H prediction. Simple Vit and ResNet18 Neural networks were trained and tested on models developed from patch-level images. A whole-slide image (WSI)-level AI model was constructed by integrating deep learning- generated pathological features with six machine learning algorithms.

**Results:**

The MLP model showed demonstrated the highest performance in predicting MSI-H in the test cohort, achieving an AUC of 93.3%, a sensitivity of 0.841, and a specificity of 0.952. Similarly, Decision Curve Analysis (DCA) revealed that WSI-level H&E-stained slides offered significant clinical MSI-H prediction in GEJ adenocarcinoma patients.

**Conclusion:**

The AI model based on digital pathology exhibits great potential for predicting MSI-H in GEJ adenocarcinoma, suggesting promising clinical applications.

## Introduction

Over the past 40 years, the incidence of gastroesophageal junction (GEJ) adenocarcinoma has steadily increased worldwide, while the incidence of gastric adenocarcinoma has been declining. As a result, GEJ adenocarcinoma is now increasingly recognized as a distinct disease entity (1–3). Due to challenges in early diagnosis, GEJ adenocarcinomas often have a poor prognosis (4). Among patients with metastatic disease, the median overall survival with optimal palliative chemotherapy is approximately 11 months (5).

Microsatellite Instability (MSI) refers to variations in length or structural abnormality of microsatellites within the genome. MSI is a critical biomarker indicating resistance to fluoropyrimidine chemotherapy and heightened sensitivity to immunotherapy. Furthermore, Microsatellite instability–high (MSI-H) GEJ adenocarcinomas are associated with better prognosis compared to microsatellite-stable tumors (6–8). According to a recent clinical trial study, Nivolumab and ipilimumab can have a high pathological response rate in the neoadjuvant treatment of tumors GEJ adenocarcinoma (9).

Pathology remains a cornerstone of tumor diagnosis. By analyzing tumor samples assessing cell morphology, structural organization, and biomarker expression clinicians can formulate tailored treatment plans. However, the accuracy of mismatch repair (MMR) immunohistochemistry, a primary method for detecting MSI, is limited by procedural quality control and antibody variability (10, 11). False negative results may prevent some patients from benefiting from immune checkpoint inhibitors, whereas false positive patients not only fail to provide treatment benefits but also expose patients to unnecessary side effects (12). Currently, traditional pathology diagnosis of MSI remains inadequate, with methods such as Multiplex Fluorescent PCR Capillary Electrophoresis or Next Generation Sequencing (NGS) with methods such as expensive and requiring specialized experimental conditions.

Deep machine learning for pathomics is an artificial intelligence (AI) technique particularly suited for processing complex visual and image data (13). Pathomics has been shown to predict tumor treatment response, tumor grade and tumor recurrence (14, 15). Rikiya’s deep learning model based on H&E histology whole-slide imaging (WSI) outperformed experienced pathologists in forecasting MSI in colorectal cancer (16). In addition to colorectal cancer, deep learning algorithms have also proven useful for detecting MSI in gastric and endometrial cancers (13, 17). Recently, xu et al. performed end-to-end training of four different tumor WSI and compared six basic models and six multi-instance learning methods to confirm that their multi-instance learning model has good application prospects in MSI prediction and clinical application (18). The end-to-end training of the multi-instance learning model was further validated for the prediction of tumor MSI. To our knowledge, MSI detection in GEJ adenocarcinoma using deep learning has not been studied to date. This study aims to construct a deep learning model to predict MSI in GEJ adenocarcinoma based on WSIs of H&E-stained histopathologic slide.

## Materials and methods

### Data collection

This study included 230 patients with GEJ adenocarcinoma admitted to the First Affiliated Hospital of Zhengzhou University from March 2021 to May 2024 were collected, 18 patients were excluded due to unclear or faded H&E staining. MSI status in all patients was determined using Multiplex Fluorescent PCR Capillary Electrophoresis or NGS. To analyze therapeutic outcomes, patients were categorized into two groups: MSI-H and MSI-L/MSS. The remaining 212 cases, represented by 416 H&E-stained slides, were randomly divided into training and testing sets at a ratio of 8:2 All HE-stained slides were scanned with the KF-PRO-005-EX digital full-slide imaging (WSI) system and exported to KFB via the K-Viewer (1.7.1.1). The first affiliated hospital of Zhengzhou university ethics committee approved the study (2023-KY-0019).

### Data processing

WSIs were divided into smaller patches measuring 512 × 512 pixels at 20 × magnification. Patches containing more than 500 pixels were selected to eliminate excessive white background, streamline subsequent processing. A notable challenge was the variation in stain color distribution among WSIs, attributed to the complexities of the staining process. To address variation in staining color distribution, the Macenko method (19) was used for slide-level color normalization. In addition, we applied Z-score normalization to the RGB channels to achieve a standard normal distribution of image intensities as input for our model. During training, online data augmentation, such as random horizontal and vertical flips, were employed. For testing, only standardization was applied. After clipping and removing the bad images, 4129722 and 1042919 patches were retained in the test set and the training set, respectively.

### Deep machine learning training

The deep learning process comprised two layers of prediction: patch-level and WSI-level. To account for diverse image sizes, WSIs were initially divided into smaller patches. A multi-instance learning algorithm was then used to aggregate patches likelihood, generating WSI-level predictions.

Patch-level predictions were generated using the widely recognized ResNet18 and Simple Vit network (20, 21), a simplified model architecture based on the Vision Transformer (ViT). This adaptation enables ViT to maintain high performance in resource-constrained environments. The primary objective was to evaluate the likelihood of each patch being accurately assigned to its corresponding WSI label.

To enhance the model’s utility across diverse cohorts, transfer learning was employed. This process involved initializing model parameters with pretrained weights from the ImageNet dataset. Patch-level discriminator weights were retained, and the entire model was subsequently fine-tuned using a limited dataset of task-specific labeled data. Through the application of transfer learning, we effectively utilized knowledge acquired from ImageNet to address our tumor classification challenge, enabling the model to perform effectively across a range of different cohorts. For the training of deep models, we used a ROG-STRIX-RTX4090 D-24G-GAMING graphics card along with an Intel 13th-generation i7-13700KF central processing, as described in the article.

After training our deep learning model, we proceeded to predict labels and their associated probabilities for all patches. These probabilities for each patch were aggregated using a classifier to generate predictions at the WSI level. In order to achieve enhanced generalization, we carefully set the learning rate by utilizing the cosine decay learning rate algorithm, and its definition is presented in the following manner.


$$\eta_{t}=\eta_{\min}+\frac{1}{2}(\eta_{\max}-\eta_{\min})\left(1+\cos\left(\frac{T_{c\text{urrent epoch}}}{T_{i}}\right)\right)$$

With the $\eta_{t}$ represents the current learning rate, $\eta_{\min}$ represents the minimum learning rate of 0, $\eta_{\max}$ represents the maximum learning rate of 0.001, $T_{i}$ represents the total number of iteration epochs= 3. Using a relatively small number of epochs is justified as our extensive dataset includes more than 5 million training patches. We also utilized transfer learning algorithms to ensure optimal model fit. The remaining parameters include optimizer -SGD, Loss function -Cross-Entropy loss and a batch-size of 32.

### Multi-instance learning for WSI fusion

Two machine learning methods, Patch Likelihood Histogram (PLH) and Bag of Words (BoW) (22), were used to consolidate patch-level predictions. The PLH method used histogram to represent the distribution of patch likelihoods across the WSI. By discretizing these likelihoods and rounding them to three decimals places, we accurately captured their distribution, enabling robust diagnostic model development. The BoW method drew inspiration from both histogram-based and vocabulary-based approaches. It utilized Term Frequency-Inverse Document Frequency (TF-IDF) (22) mapping for individual patches, creating TF-IDF feature vectors that summarized the entire WSI. These feature vectors were subsequently used to train conventional machine learning classifiers to predict the MSI status for each WSI.

### Transformer based feature fusion

By integrating these two pipelines, we consolidated initially fragmented patch-level predictions into comprehensive WSI level features. These enriched features significantly enhance the downstream analytical processes. At the same time, Based on the cross-attention of transformer algorithm, histograms and TF-IDF features were fused by dynamic weight allocation and semantic space alignment to construct the transformer model.

### Signature building

Patient representations in this study were constructed by integrating patch-level predictions, probability histograms, and TF-IDF features. A t-test statistical analysis was initially employed to identify significant pathological features, refining the feature selection process for both diagnostic models. To build robust prediction models, 5-fold cross-validation was applied to the training set and a range of machine learning algorithms were used, including support vector machine (SVM), tree-based models such as random forest, gradient boosting methods such as extreme gradient boosting (XGBoost) and optical gradient boosting machine (LightGBM). In addition, we incorporated multilayer perceptron (MLP) and logistic regression (LR) into our modeling framework. We selected the best performing hyperparameter combination based on grid-search by five-fold cross validation. The hyperparameters of the six machine learning models are as **Supplementary Table 1**.

### Model evaluation

The ability of the model to accurately predict MSI-H was evaluated using the ROC curve at the patch level. To further assess performance, we visualized the aggregation of patch predictions into WSI. Predicted labels and probability heatmaps were generated to facilitate detailed analysis. For performance metrics, we used AUC and calculated sensitivity and specificity to comprehensively assess the prediction model’s efficacy. In this study, a variety of software tools were utilized, including ITK SNAP v.3.8.0, custom Python code written in Python v.3.7.12. The Python packages used for analysis included Pandas v.1.2.4, NumPy v.1.20.2, PyTorch v.1.8.0, Onekey v.2.2.3, OpenSlide v.1.2.0, Seaborn v.0.11.1, Matplotlib v.3.4.2, SciPy v.1.7.3, Scikit-learn v.1.0.2, and PyRadiomics v.3.0.

## Results

### Clinical characteristics of patients with GEJ adenocarcinoma

**Figure 1:**
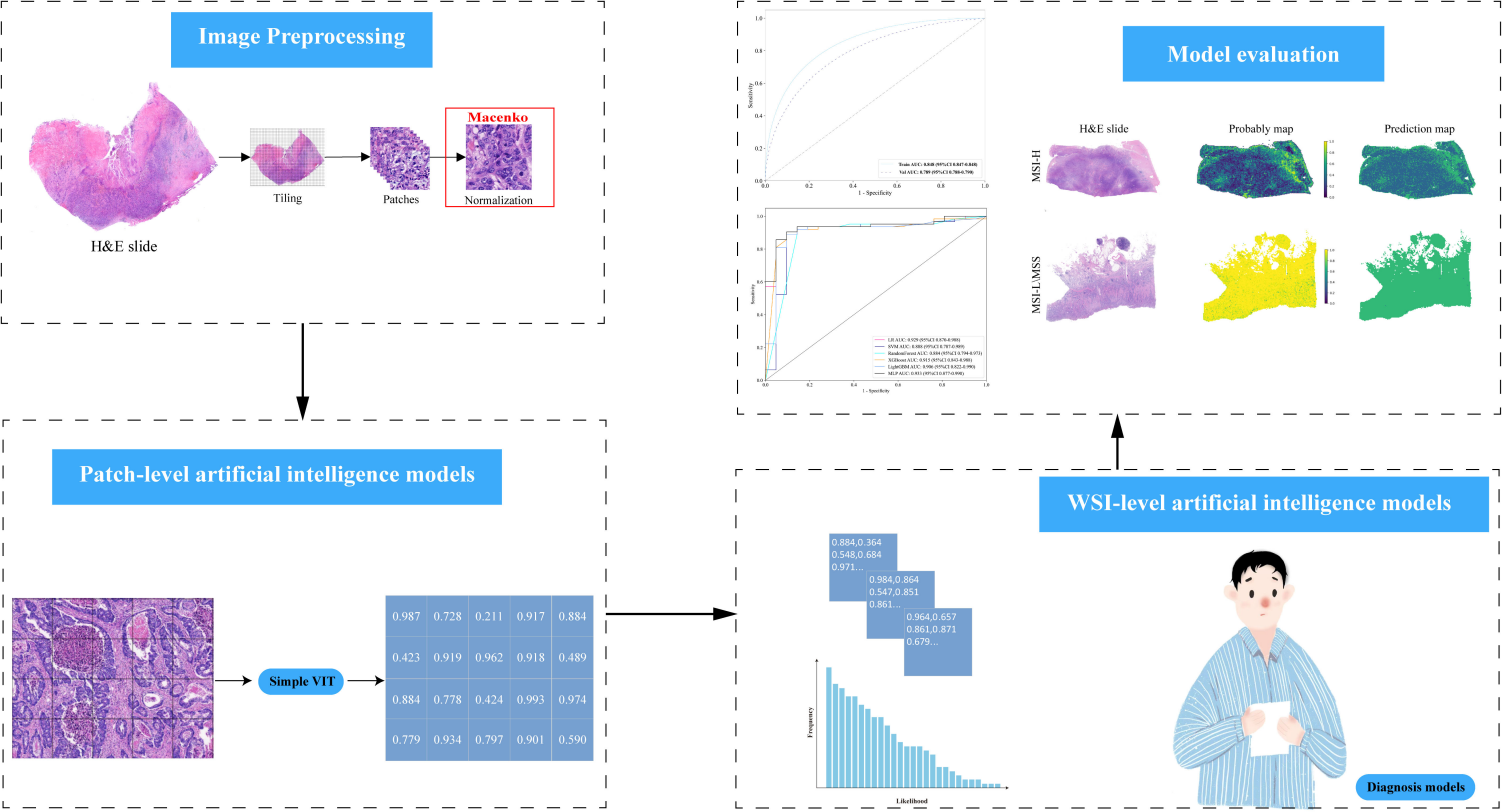
Workflow Diagram for AI Model Development: Gather H&E slides from GEJ adenocarcinoma patients and use Simplevit to create a patch-level AI model. Implement six machine learning techniques to develop WSI-level AI models, and assess the performance of each model on the test dataset.

A total of 212 patients diagnosed with EGJ adenocarcinoma through PCR or NGS between March 2021 and May 2024 were retrospectively included in this study from the First Affiliated Hospital of Zhengzhou University. WSIs was performed on 416 H&E-stained slides from these patients. The slides were randomly allocated into training (332 slides) and validation (84 slides) in a 8:2 ratio. **Table 1** provides a summary of the patients’ clinical characteristics, and the test flowchart is shown in **Figure 1**. No significant differences were observed between MSI-H and MSI-L/MSS patients concerning smoking history, alcohol consumption, T stage, metastasis in the liver, bone, or brain. In terms of gender, we found that the MSI-H above the MSI-L/MSS patients (P<0.01).

**Table 1:** Baseline characteristics of patients in MSI-H and MSI-L\MSS.

Patient characteristics	MSI-H	MSI-L\MSS	P
Age	64.88 ± 8.81	60.19 ± 10.03	0.01
Sex			0.491
Male	28 (70.00)	132 (76.74)	
Female	12 (30.00)	40 (23.26)	
Smoking history			0.619
Yes	9 (22.50)	48 (27.91)	
No	31 (77.50)	124 (72.09)	
Drinking history			0.729
Yes	7 (17.50)	37 (21.51)	
No	33 (82.50)	135 (78.49)	
T grade			0.731
1	2 (5.00)	15 (8.72)	
2	8 (20.00)	28 (16.28)	
3	26 (65.00)	105 (61.05)	
4	4 (10.00)	24 (13.95)	
Pulmonary metastasis			1
Yes	0	2 (1.16)	
No	40 (100.00)	170 (98.84)	
Osseous metastasis			1
Yes	0	2 (1.16)	
No	40 (100.00)	170 (98.84)	
Hepatic metastases			1
Yes	2 (5.00)	11 (6.40)	
No	38 (95.00)	161 (93.60)	
Brain metastases			1
Yes	0	1 (0.58)	
No	40 (100.00)	171 (99.42)	

### t-SNE visualization

**Figure 2:**
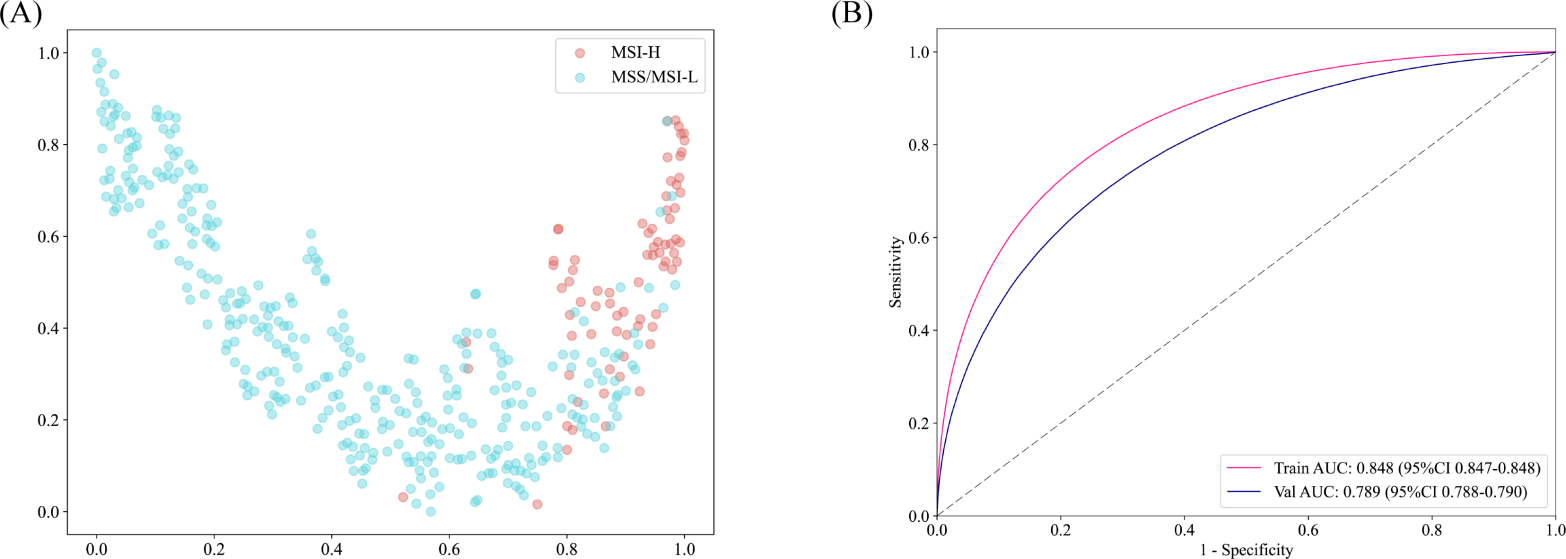
**(A)** Plotting the t-SNE algorithm for MSI-H and MSS/MSI-L in a two-dimensional space. **(B)** The patch-level AUC for predicting MSI-H, MSS, and MSI-L in the training and test cohorts by Simple ViT model.

To facilitate-class classification in the diagnostic model, feature dimensionality was reduced to single decimal places. The t-SNE algorithm was employed to visualize how patch-level features aggregated into WSI representations (**Figure 2A**). This approach revealed a clear separation between the MSI-H and MSI-L/MSS groups when visualized in a two-dimensional space. The Grid-Search algorithm was utilized to identify optimal model parameters, which were subsequently fine-tuned through five-fold cross-validation.

### Deep learning and machine learning efficiency

**Figure 3:**
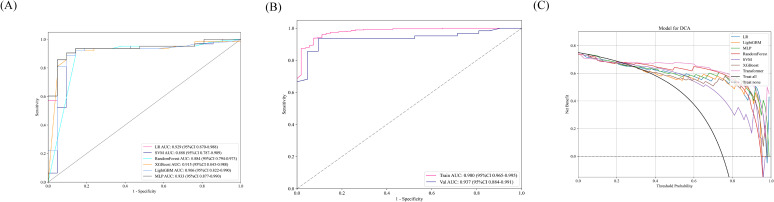
**(A)** In the test cohort, the WSI-level AUCs of the prognostic model across six different machine learning models. **(B)** AUC of the transformer model training and test sets at the WSI level. **(C)** The DCA curve indicated that the MLP model could also obtain good benefits.

The accuracy of the pathology model in identifying MSI-H was assessed using patch-level ROC curves for detailed model characterization (**Figure 2B**, **Supplementary Figure 1**). At the patch level, the Simple Vit (0.789) algorithm had a higher AUC than the ResNet (0.638) in test cohort. Furthermore, the higher model power for predicting MSI-H at the WSI level indicates a significant improvement in feature modeling when aggregating using BoW and PLH methods. These findings underscore the effectiveness of our feature aggregation approach. Among the tested machine learning approaches, the MLP algorithm demonstrated the most accurate classification results on the test cohort, as indicated by the AUC (**Figure 3A**, **Supplementary Figure 2A**). However, the transformer model showed higher AUC compared with the six machine algorithms in test cohort, highlighting its advantage in prediction performance (**Figure 3B**), and delong’s test was used to compare the AUCs of the six machine learning algorithms and the transformer model (**Supplementary Figure 2B**). Unfortunately, there was no significant difference. AUC, specificity, and sensitivity values for the training and test cohorts across all seven models are presented in **Supplementary Table 2**. Additionally, confusion matrices of transformer model for test cohorts were generated to visually illustrate classification performance (**Supplementary Figure 3**).

### Decision curve analysis and model interpretability

**Figure 4:**
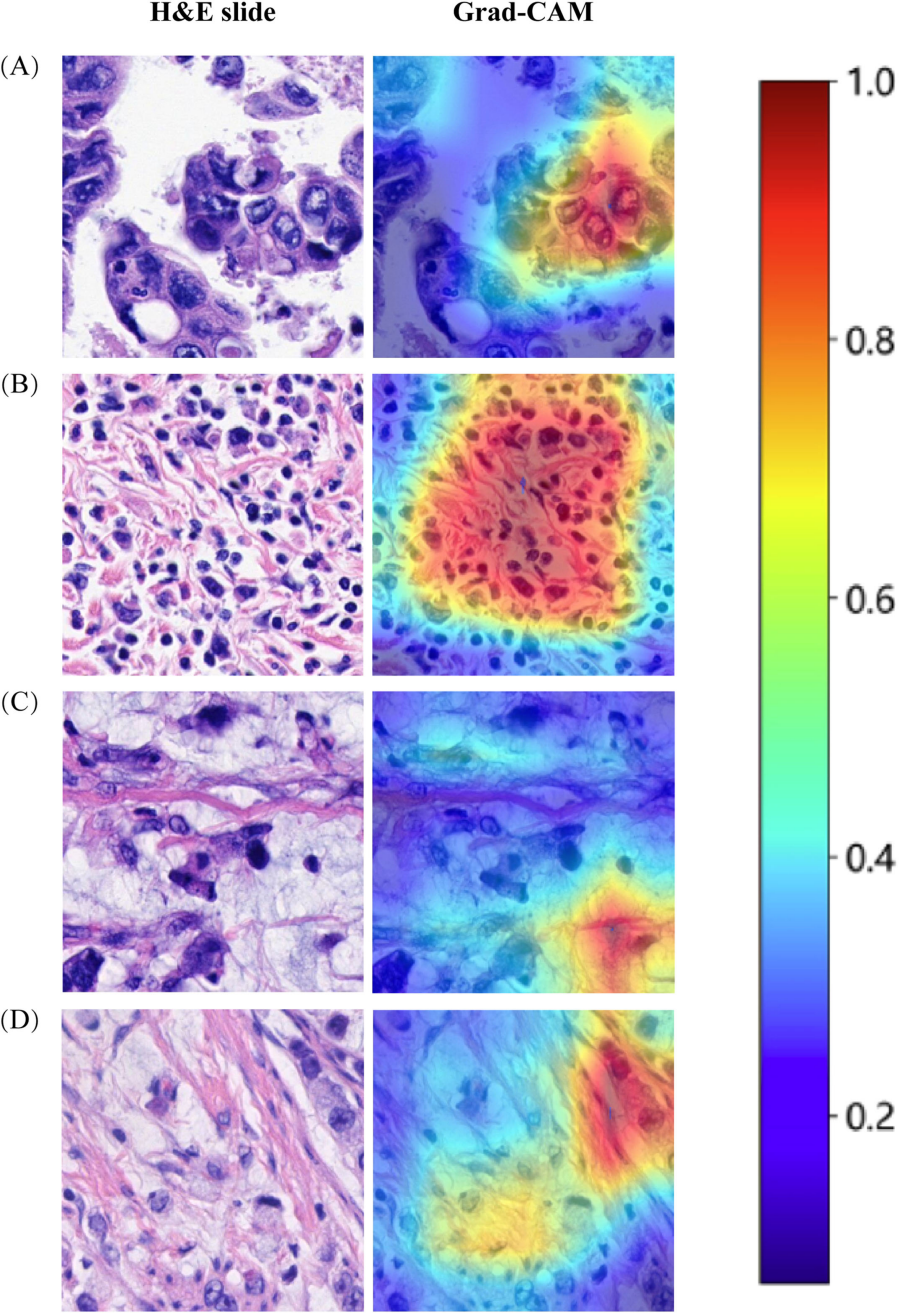
The use of Grad-CAM to visualize the activation of the diagnostic model. **(A)** Highly pleomorphic tumor cells. **(B)** Numerous tumor-infiltrating lymphocytes. **(C)** Mucinous adenocarcinoma. **(D)** Signet ring cell carcinoma.

**Figure 5:**
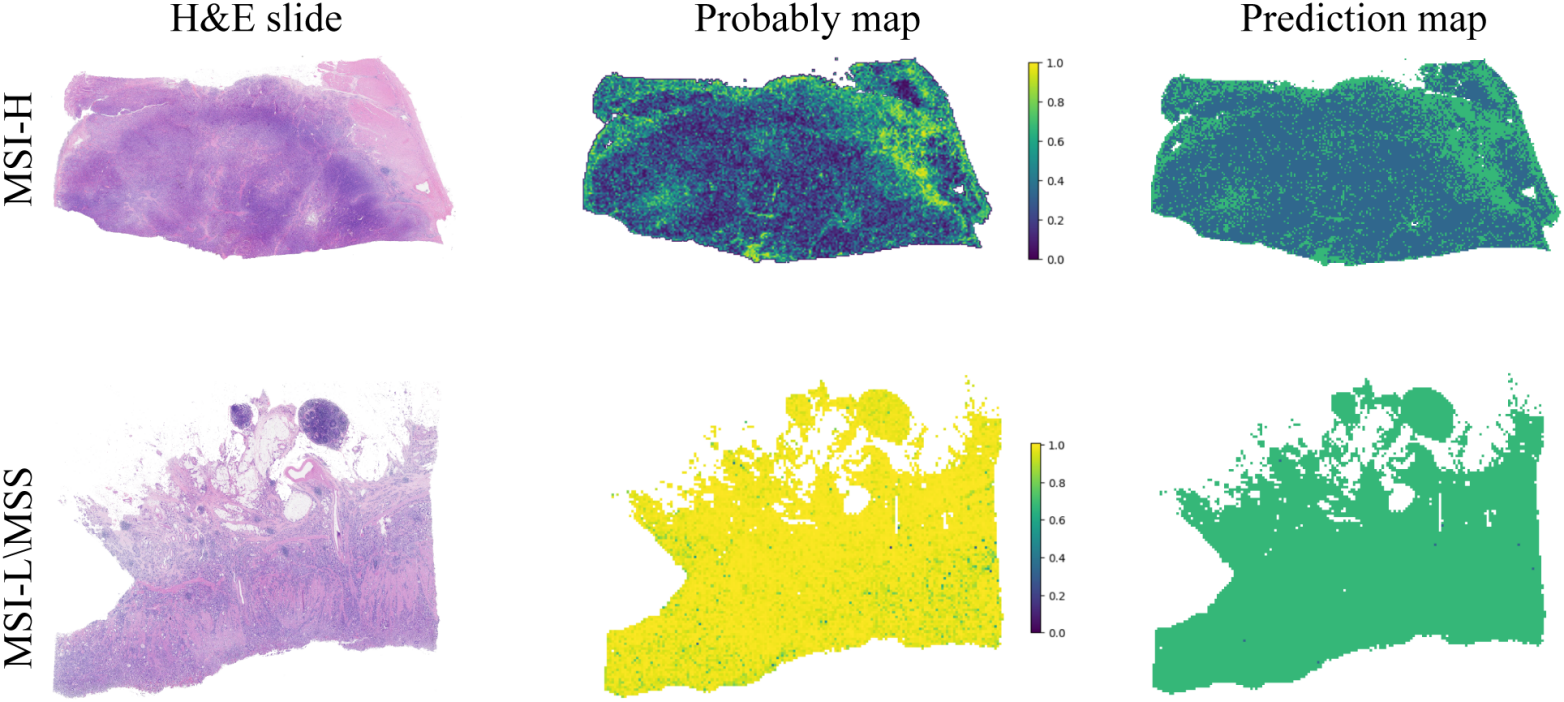
The diagnostic model’s probability and prediction heatmaps are displayed. On the left, the figure shows the WSI-level H&E slides. In the middle, it presents the heatmap of predicted probabilities for each patch. On the right, the prediction results for the WSI are shown.

Decision Curve Analysis (DCA) based on the seven model is shown in **Figure 3C**. To further interpret the model’s decision-making, Grad-CAM heatmaps were employed. These heatmaps visually highlight areas of significant neural network during classification, with darker regions indicating stronger contributions to predictions. Importantly, Grad-CAM retains spatial information for each class without requiring modifications or additional training. **Figure 4** demonstrates Grad-CAM ability to decode feature map importance by analyzing gradients in the last convolutional layer. This transparent visualization identifies input regions with the highest impact on predictions, offering valuable insights into the model’s interpretability. The red heat map highlights the highly pleomorphic tumor cells and the large number of tumor-infiltrating lymphocytes. Interestingly, the red heat map also highlights the mucus in the interstitium as well as signet ring cells with large amounts of mucus inside the cytosol. These features have been suggested to be associated with MSI-H in previous studies. Finally, the probability and prediction heatmaps generated by pathology model (**Figure 5**) demonstrate its high accuracy in assessing region tiles, further validating its robust performance.

## Discussion

Over the past five decades, the global incidence of tumor increased gradually, imposing a significant economic burden on healthcare systems worldwide (2, 23–26). The prognosis for GEJ tumors remain poor, with 5-year survival rates for early-stage cases rarely exceed 25% to 35% (27). GEJ cancer can be divided into four subgroups according to their molecular characteristics: MSI tumors, Epstein-Barr virus-infected tumors, genome stable tumors, and chromosome instability tumors (28). MSI tumors are further categorized into MSI-H, MSI-L, and MSS on mutation frequency. MSI-H tumors are characterized by increased lymphocyte infiltration and high PD-L1 expression, making them promising candidates for immunotherapy in patients with MSI-H-related GEJ adenocarcinoma (29). Accurate detection of MSI status in GEJ adenocarcinoma is therefore critical, particularly given its role in predicting response of immune checkpoint inhibitors and the high pathological complete response rates seen with neoadjuvant therapy in resectable MSI-H cases (9, 30). Therefore, MSI accurate detection for GEJ adenocarcinoma patients is very important.

Computational pathology (CP) combines AI and machine learning, leveraging digital pathomics to extract information beyond what the human eye can perceive. CP has been applied in the routine pathological diagnoses, predicting the treatment outcomes for patients, and discovering molecular markers (13, 31, 32). Conventional histopathology remains the gold standard for cancer diagnosis, placing significant responsibilities on pathologists. They need to diagnose and evaluate the disease while providing prognostic information, including disease classification and grading. However, these decisions rely on intricate visual characteristics and require extensive expertise and training (33). For young pathologists, the primary challenge is managing heavy clinical workloads while contending with limited professional knowledge. The application of CP can alleviate their workload and facilitate more accurate diagnosis. In addition, CP can standardize processes such as image acquisition, analysis, interpretation and reporting, addressing issues that arise during the diagnosis process (31).

In a study, a DL prediction model was constructed from H&E WSIs of 50 MSS and 50 MSI-H colorectal cancer cases. The area under the receiver operating characteristic curve of the model’s test set was significantly higher than that of the five pathologists (16). Interestingly, Kather et al. have shown that a trained classifier on the gastric carcinoma underperforming in colorectal cancer (13). Additionally, Lee et al. demonstrated that classifiers trained on colorectal cancer also performed poorly on gastric carcinoma (34). These two studies show that the characteristics of gastric carcinoma and colorectal carcinoma MSI are incompatible, consistent with the morphological differences observed in MSI-H between gastric carcinoma and colorectal carcinoma. By the same token, GEJ adenocarcinomas differ from esophageal and gastric carcinoma in terms of etiology, pathogenesis and natural history, and have been regarded as a unique disease entity. Therefore, there is an urgent need to develop AI technologies based on digital pathology for the prediction of GEJ adenocarcinoma.

In recent years, CP has made remarkable progress in the field of MSI prediction, particularly with Transformer architecture-based DL models, such as MSIscope, achieving high accuracy and rapid detection through multiscale feature fusion (35, 36). These techniques are not only suitable for colorectal cancer, but also provide new ideas for MSI-H prediction of GEJ adenocarcinomas. In this study, we developed a DL model based on multi-instance learning and transformer algorithm to assist pathologists in determining MSI-H in GEJ adenocarcinoms based on h&e slides. In the future, the development of automated tools in combination with prospective clinical trials, such as the immunotherapy cohort recommended by the NCCN guidelines, is expected to drive the clinical translation of computational pathology in GEJ adenocarcinomas.

At present, in addition to deep learning (DL) models based on H&E staining at the section level to predict tumor MSI-H expression, there are also DL methods based on radiomics features for predicting MSI expression. Jiang et al. (37) extracted radiomics features from pretreatment contrast-enhanced CT images of 223 gastric cancer patients, and build the clinical model, radiology, and hybrid model to predict the MSI expression. Although their study achieved high accuracy, the MSI expression levels in their patients were based on immunohistochemistry of pathological sections, which is subject to some false negatives. In contrast, the patients in our research had their MSI levels detected using PCR or NGS, ensuring the accuracy of the model building. Hu et al. (38) developed a deep-learning model based on weakly supervised learning to predict MSI status in prostate cancer patients and evaluated its generalizability on externally stained and scanned slides as well as in a time-independent validation cohort. In addition, Wang et al. (39) predicted MSI expression levels based on H&E-stained sections in endometrial cancer. These studies suggest MSI expression can be predicted MSI expression with high accuracy in a variety of solid tumors based on H&E staining. In addition, only a few MSI-H and MSI-L\MSS cases were not separated according to the t-SNE dimension reduction results, which further shows the reliability and stability of the prediction model. The results of the DCA curve demonstrate that our model brings greater benefits in predicting MSI-H in GEJ adenocarcinoma patients.

Our research has some limitations. First, this study only investigated samples from a single center. In the future, we plan combine multiple centers as an external validation set to verify the performance of our model. Second, similar to several published studies predicting MSI expression levels (37, 38, 40), the proportion of MSI-H patients in our study was low, which is consistent with the low proportion of MSI-H patients in total GEJ adenocarcinomas.

In conclusion, we developed a predictive model for MSI-H based on digital pathology using the H&E-stained slides of 212 GEJ adenocarcinoma patients. The model demonstrated good performance in both the test and validation datasets.

## Data Availability

The original data on which the conclusions of this article are based can be obtained by contacting the corresponding author.

## Supplementary material

Supplementary Figure 1 The patch-level AUC for predicting MSI-H, MSS, and MSI-L in the training and test cohorts by ResNet18 model.

Supplementary Figure 2
**(A)** In the training cohort, the WSI-level AUCs of the prognostic model across seven models. **(B)** Delong test results for six different machine learning model and transformer model.

Supplementary Figure 3 Confusion matrix for the WSI-level transformer model. The 0 on the ordinate and abscissa refers to MSI-L/MSS.;1 represents MSI-H.
