# Supplementary material for: Digital pathology-based artificial intelligence model to predict microsatellite instability in gastroesophageal junction adenocarcinomas
Source: Front Oncol. 2025 Aug 7;15:1486140. doi: 10.3389/fonc.2025.1486140 (PMC12367487; doi:10.3389/fonc.2025.1486140)
Supplement: Supplementary file 1 [file Table1.docx]

Supplementary Table1 Parameters of machine learning.

| Classification | Parameters |
| --- | --- |
| LR | penalty = 'none', max_iter = 100 |
| SVM | probability = True, max_iter = 100, kernel = 'linear' |
| RandomForest | n_estimators = 5, max_depth = 3, min_samples_split = 4, random_state = 0 |
| XGBoost | n_estimators = 6, objective = 'binary:logistic', max_depth = 3, min_child_weight = 0.2, use_label_encoder = False, eval_metric = 'error' |
| LightGBM | n_estimators=2, max_depth=1, min_child_weight = 0.5 |
| MLP | hidden_layer_sizes = (61, 128, 64, 32), max_iter=300, solver='sgd', random_state=0 |
