# Supplementary material for: Digital pathology-based artificial intelligence model to predict microsatellite instability in gastroesophageal junction adenocarcinomas
Source: Front Oncol. 2025 Aug 7;15:1486140. doi: 10.3389/fonc.2025.1486140 (PMC12367487; doi:10.3389/fonc.2025.1486140)
Supplement: Supplementary file 2 [file Table2.docx]

Supplementary Table 2 Performances of the nominated binary classifiers in training cohort and test cohort

| Classification | Training cohort | | | Test cohort | | |
| --- | --- | --- | --- | --- | --- | --- |
|  | Sensitivity | Specificity | AUC | Sensitivity | Specificity | AUC |
| LR | 0.853 | 0.963 | 0.971 | 0.841 | 0.952 | 0.929 |
| SVM | 0.939 | 0.944 | 0.971 | 0.889 | 0.905 | 0.888 |
| RandomForest | 0.978 | 0.981 | 0.999 | 0.889 | 0.857 | 0.884 |
| XGBoost | 0.989 | 0.981 | 0.998 | 0.889 | 0.905 | 0.915 |
| LightGBM | 0.917 | 0.944 | 0.984 | 0.857 | 0.905 | 0.906 |
| MLP | 0.835 | 0.981 | 0.97 | 0.841 | 0.952 | 0.933 |
| Transformer | 0.935 | 0.926 | 0.980 | 0.921 | 0.905 | 0.938 |
