# Supplementary figures and images for: Digital pathology-based artificial intelligence model to predict microsatellite instability in gastroesophageal junction adenocarcinomas
Source: Front Oncol. 2025 Aug 7;15:1486140. doi: 10.3389/fonc.2025.1486140 (PMC12367487; doi:10.3389/fonc.2025.1486140)

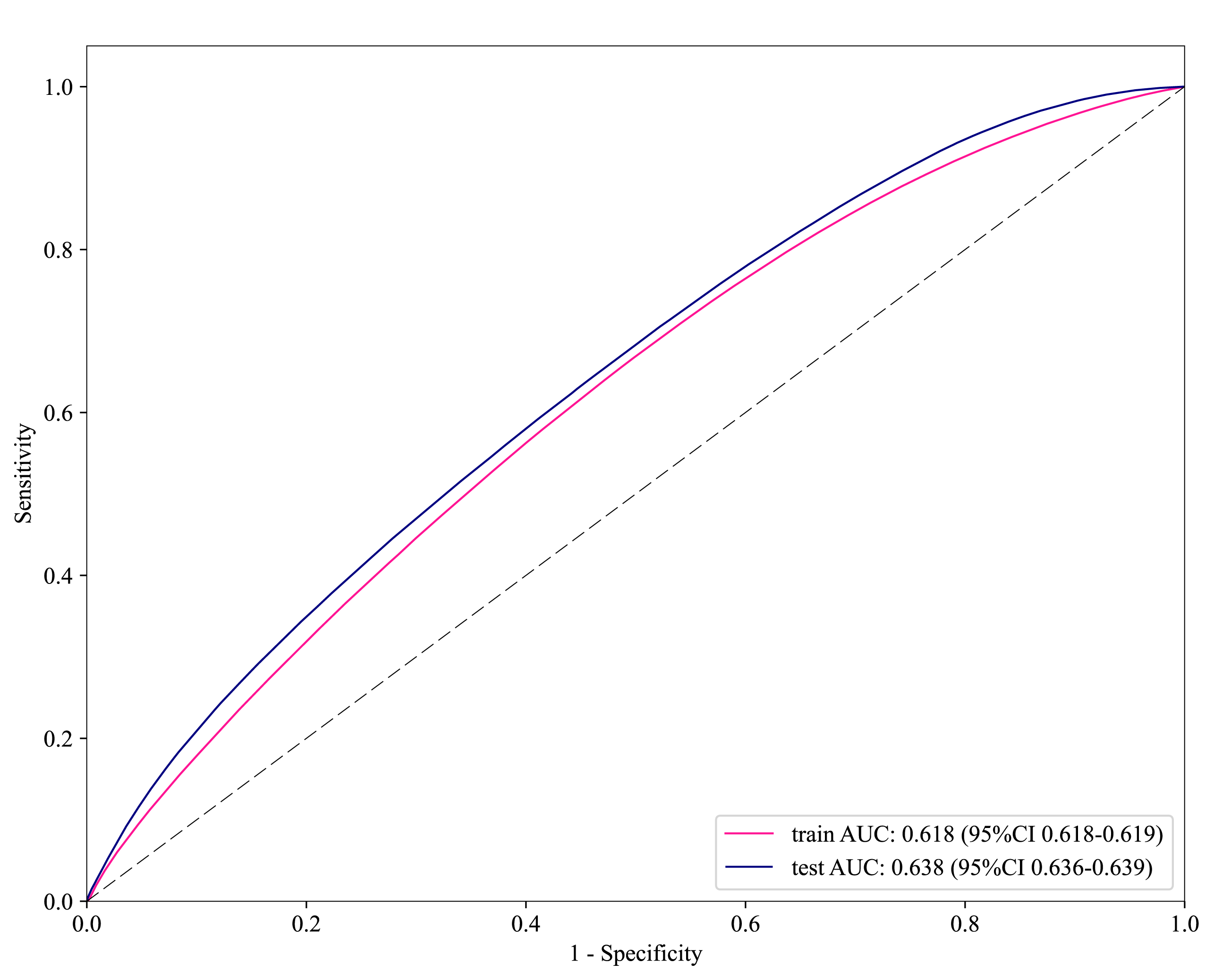

Supplement: Supplementary Figure 1 — The patch-level AUC for predicting MSI-H, MSS, and MSI-L in the training and test cohorts by ResNet18 model. [file Image1.tif]

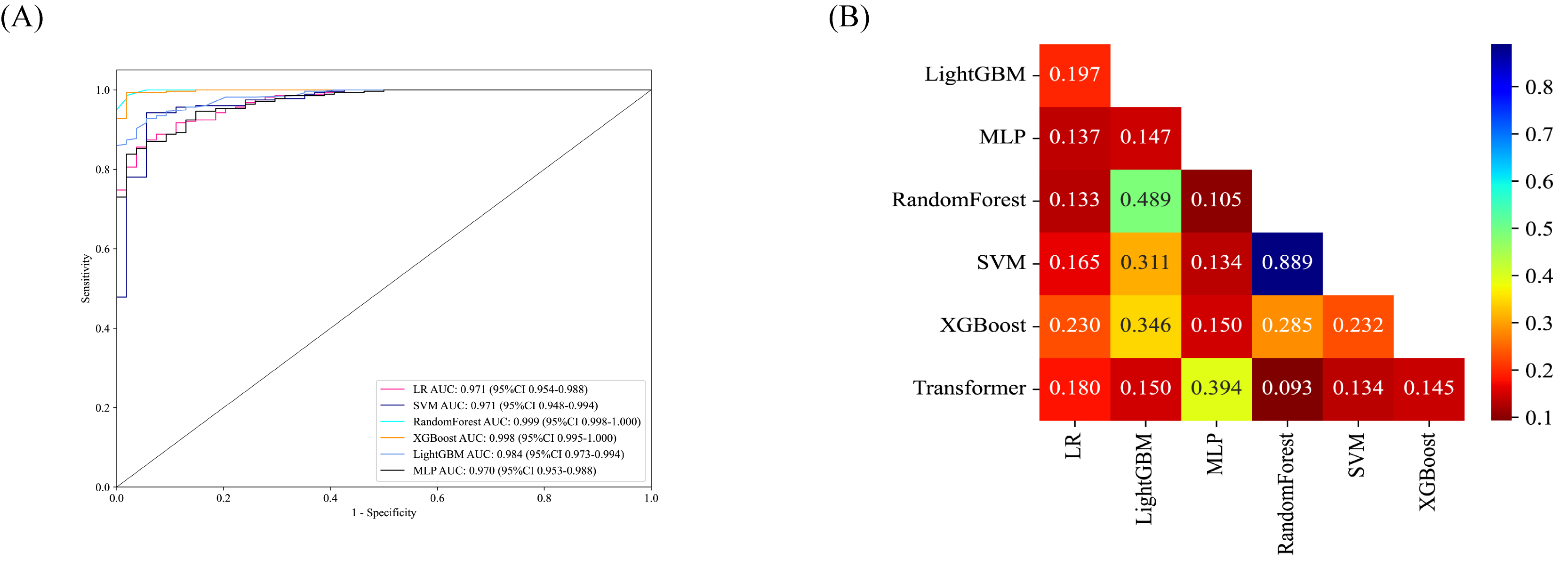

Supplement: Supplementary Figure 2 — (A) In the training cohort, the WSI-level AUCs of the prognostic model across seven models. (B) Delong test results for six different machine learning model and transformer model. [file Image2.tif]

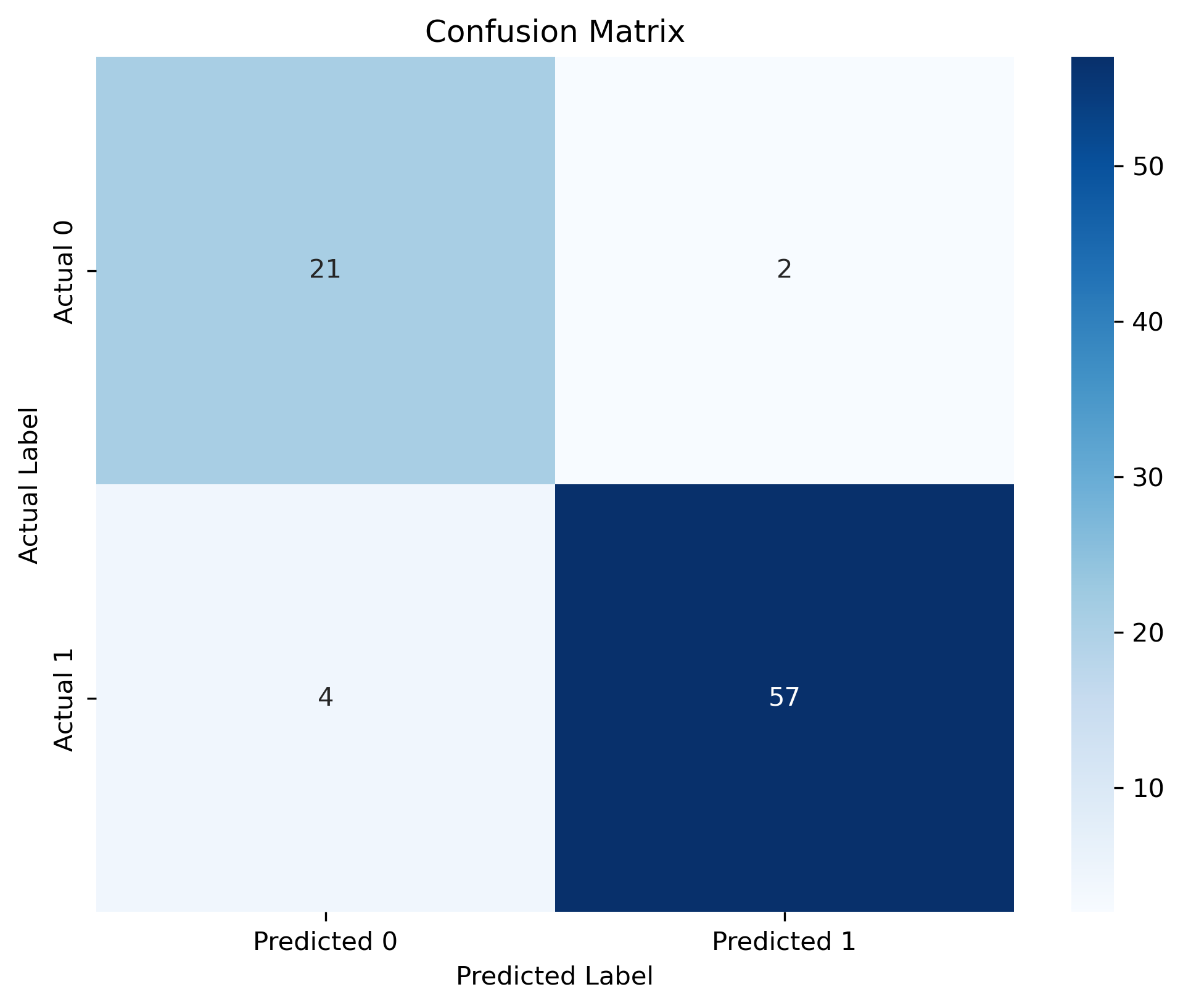

Supplement: Supplementary Figure 3 — Confusion matrix for the WSI-level transformer model. The 0 on the ordinate and abscissa refers to MSI-L/MSS.;1 represents MSI-H. [file Image3.png]
